# Fertile Grounds of Inquiry: Environmental Effects on Human Reproduction

**DOI:** 10.1289/ehp.114-a644

**Published:** 2006-11

**Authors:** Julia R. Barrett

In a world whose population exceeds 6.5 billion, declining human fertility might not seem to be a critical problem. After all, overpopulation has been a global concern for decades. Declining fertility rates in more advanced nations largely reflect the changing role of women and their rapidly growing presence in the workplace—fertility declines may stem at least in part from the modern tendency to delay child-bearing until later in life, when fertility naturally declines. But this doesn’t explain the fact that, according to a December 2005 report of the CDC’s National Survey on Family Growth (NSFG), the fastest-growing segment of U.S. women with impaired fecundity (the capacity to conceive and carry a child to term) is those under 25. The rising incidence of fertility-impairing health factors such as obesity also likely plays an important role. Clues from environmental exposure assessments, wildlife studies, and animal and human studies hint at additional factors: exposure to low-level environmental contaminants such as phthalates, polychlorinated biphenyls (PCBs), dioxins, pesticides, and other chemicals may be subtly undermining our ability to reproduce.

As recognized by the American Society of Reproductive Medicine, infertility is a biological disease that impairs a couple’s ability to achieve a viable pregnancy. It can be caused by hormonal, ovarian, uterine, urological, and other medical factors. Known risk factors include advanced age, being over-or underweight, lack of exercise, smoking, alcohol and substance abuse, sexually transmitted diseases, and poor nutrition.

According to the American Society of Reproductive Medicine, a medical infertility cause can be identified, or perhaps only indefinitely suggested, in approximately 90% of cases and may be multifactorial in 25% of cases. Male factors include low sperm count and sperm abnormalities, such as altered morphology and low motility. Female factors stem from ovulation problems such as premature ovarian failure (early menopause), thyroid irregularities, polycystic ovarian syndrome, and fallopian tube obstruction.

Up to 10% of infertility cannot be explained medically. Fertility transcends the reproductive system, notes Louis Guillette, a professor of zoology at the University of Florida in Gainesville. “When you talk about infertility, you literally are talking about probably almost every system in the body—infertility is an integrated signal of all these different systems,” he explains. “Trying to tease out which system, or more than likely what multiple systems have been altered, leading to that phenomenon, is very tough work.”

Infertility is generally defined as occurring when a couple cannot become pregnant after trying to conceive for at least one year (or six months if the woman is over age 35). According to the 2001 WHO report *Current Practices and Controversies in Assisted Reproduction*, at least 80 million people worldwide are estimated to be affected by infertility. Infertility rates range from less than 5% to greater than 30% depending on location and how infertility is defined, with higher rates associated with lack of medical care access. Based on the 2005 NSFG report, approximately 12% of American couples experienced impaired fecundity in 2002. This is a 20% increase from the 6.1 million couples who reported an inability to have children in 1995.

Determining whether infertility is actually increasing is more complicated than these numbers imply, however. In a paper published in the September 2006 issue of *Fertility and Sterility*, David Guzick and Shanna Swan of the University of Rochester School of Medicine and Dentistry noted that “impaired fecundity” as defined by the NSFG implies a decrease in fertility, but the same study also showed that fertility, defined there as a married woman unable to become pregnant within 12 months, has increased.

The absence of definitive information can frustrate couples experiencing fertility problems as well as experts. “There seems to be more to it than can be explained from traditional understanding about impacts,” says Joseph Isaacs, president and CEO of RESOLVE: The National Infertility Association. “As a patient advocacy group, we believe more research into environmental impacts is needed. We fear that future generations may be at risk because of exposures to toxic substances as early as *in utero*.”

## Foundations of Fertility

A person’s reproductive potential begins shortly after his or her own conception. Based on the embryo’s chromosomal inheritance, hormonal signals are created to direct the structure and function of the reproductive tract. Normal development depends upon a correct balance of androgen and estrogen signals being delivered at appropriate times.

Fetal development can be altered by external factors as demonstrated by the human experience with the synthetic estrogen diethylstilbestrol (DES), prescribed to prevent miscarriage between 1947 and 1971. The drug didn’t affect mothers, and it didn’t lower miscarriage incidence; in fact, it significantly increased it. It also induced changes in the developing reproductive tract of female offspring.

In the 15 April 1971 issue of the *New England Journal of Medicine*, it was reported that daughters with prenatal DES exposure had significantly increased incidence of vaginal cancer, which is normally quite rare and was virtually unknown in young women prior to DES. Later research revealed structural abnormalities of these women’s reproductive tracts and effects in their male offspring including increased risk of cryptorchidism (undescended testes) and low sperm counts.

The study of endocrine disruptors has raised concerns about the reproductive effects of exposure to certain environmental compounds that affect the endocrine system via estrogenic, androgenic, antiandrogenic, and antithyroid mechanisms. One key report was a 12 September 1992 review in the *British Medical Journal* indicating significant declines in sperm counts in many countries between 1938 and 1990. The findings were controversial because the reviewed studies used inconsistent designs and methods. In October 1997, however, a review published in *EHP* by Swan and others confirmed the findings for males in the United States and indicated an even sharper decline among European men. Other studies have found declines for specific areas or no decline at all.

“I think the evidence across studies is mixed,” says Russ Hauser, an associate professor of environmental and occupational epidemiology at Harvard School of Public Health. “Historical studies were not designed to explore this question. It wasn’t that someone set out forty or fifty years ago to design a study to look at how semen quality is going to change over time.” There are going to be limitations in the data because of that, he explains, so it’s hard to determine whether there is a true temporal trend. “However,” he adds, “the data suggest there are definite geographical differences between countries and regions within countries in semen quality.”

According to Niels Skakkebæk of Rigshospitalet in Copenhagen and colleagues writing in the February 2006 issue of the *International Journal of Andrology*, comparisons of sperm quality among populations of European men have revealed that as many as 30% of young Danish men have low sperm count, and an additional 10% may be infertile. Denmark also has an unusually high rate of testicular cancer. Rates have been increasing in many countries over the last 50 years, but the Danish rate is noticeably higher; for example, four to five times higher than the Finnish rate.

This difference prompted researchers to also examine incidence of hypospadias (in which the urethra opens along the underside of the penis shaft rather than the tip) and cryptorchidism. Not only did both disorders occur more frequently in Danish boys compared with Finnish boys, but the Danish rates had risen in recent decades. These findings as a whole inspired Skakkebæk and colleagues to propose, in the May 2001 issue of *Human Reproduction*, an overarching disorder, testicular dysgenesis syndrome (TDS), in which perturbation of testis development in fetal life sets the stage for hypospadias, cryptorchidism, testicular cancer, and reduced sperm quality.

It’s reasonable to suspect there might be a female corollary to TDS. “We have no really good reasons not to expect that women are as sensitive to environmental chemicals as the males are,” says Jens Peter Bonde, a professor of occupational medicine at Århus University Hospital in Copenhagen. He points out that it’s easier to study male fertility because men can easily provide sperm samples. “That’s one basic reason that there has been so much attention on the males, but from a biological point of view one would definitely expect that the female reproductive system might be vulnerable also,” says Bonde.

According to Guillette, another stumbling block is the accepted, but unproven, dogma that an embryo will develop as a normal female barring any hormonal signals to become male. “It hasn’t been an area where there have been substantial amounts of work done. There’s certainly very good work, but not the same kind of huge body of literature that one sees about the developing testis and the male reproductive system,” he says.

One of the few epidemiologic studies to link low-level human exposure to an environmental contaminant with a specific end point was Swan and colleagues’ investigation of prenatal phthalate exposure, published in the August 2005 issue of *EHP*. Their results suggested a subtle change in boys’ development—a shortening of the anogenital index (the distance between the anus and the scrotum, divided by weight)—associated with prenatal exposure to several phthalates. This finding is not a predictor of future fertility and needs confirmation, but it is noteworthy as the first study to link verified prenatal exposure to a specific outcome.

## Animal Findings to Human Concerns?

Consequences of disrupting the normal hormone milieu have also been observed in wildlife. Examining alligators in polluted lakes in northern Florida, Guillette’s group has observed altered function of the ovaries and testes, smaller penis size, and abnormalities that extend to the thyroid gland, liver, and immune system. A robust body of literature details reproductive effects in fish, amphibians, and reptiles related to their exposure to endocrine disruptors. Evidence of these effects has also been seen in wild mammals such as polar bears and seals. Laboratory animal experiments have confirmed these wildlife findings, demonstrating that effects are not necessarily from steroid receptor disruption, however, but may, for example, be observed in altered synthesis and control of endogenous hormones.

The study of fertility also encompasses pregnancy, especially the early weeks following fertilization. Early pregnancy loss is normally quite high in humans, with an estimated 30% of pregnancies ending in miscarriage in the first six weeks. A frequent cause of miscarriage is aneuploidy, an incorrect number of chromosomes in the embryo, and mouse studies have shed some light on potential environmental contributors to this condition.

During a 1998 investigation of age-related aneuploidy rate increases, Patricia Hunt, a professor of molecular biosciences and a reproductive biologist at Washington State University, and her colleagues were amazed to see a sudden rate spike in their mouse colony. An investigation revealed correlation between damage to the plastic mouse cages and the chromosomal abnormality. Further scrutiny implicated bisphenol A (BPA), a suspected environmental estrogen used in plastics manufacture, as the potential causal agent. In a study published in the 1 April 2003 issue of *Current Biology*, the researchers replicated exposure experimentally and found that BPA derailed proper chromosome segregation during oocyte meiosis.

An extension of this research has been completed with amazing—but not yet published—results, and Hunt hopes that the line of inquiry can be extended to humans. “One of the things that my new research on BPA has made me wonder is whether or not there could be environmental effects that would change the frequency or in specific populations might cause noticeable differences in aneuploidy,” she says.

Hunt says it’s hard to know precise numbers of human aneuploidy cases. “We can’t see the loss that occurs preimplantation, but we make an assumption that there’s quite a bit, based on what we can see and what we think must happen,” she says. But whether there’s been an increase in aneuploidy over time cannot be known. “Human aneuploidy studies were done mostly in the 1970s and early 1980s,” says Hunt. “Is this aneuploidy rate the same across all populations? To the best of our knowledge, it has been, at least in those previous studies. But is the rate the same as it was then? We wouldn’t know. We wouldn’t be able to see a dramatic increase in chromosomally abnormal spontaneous abortions, because those kinds of studies aren’t currently under way.”

Extending animal studies to human health is a challenge, though. Genetically, the reproductive system is highly conserved across species, making it likely that responses to inputs would be similar. But species differences in exposure, metabolism, and anatomy preclude making a direct comparison.

“Wildlife studies cannot be related to humans one to one,” says Guillette. “If one’s looking at the functioning of the ovary, or the functioning of the brain, and hormones, and even the genes that seem to be involved with the proliferation or the growth of the uterus or the development of an egg, for example, they’re incredibly conserved.” He explains that if problems are seen in these animals at a certain level, and researchers are able to identify mechanisms that are being disturbed leading to those abnormalities, then that raises possible concerns for humans, even if humans are exposed in a slightly different manner.

## Worldwide Concerns

Geographic differences may suggest environmental exposures that need investigation, wrote Swan in a paper published in the February 2006 issue of *Seminars in Reproductive Medicine*. For example, in the first phase of the EPA-funded Study for Future Families, of which Swan is the principal investigator, she and her colleagues saw significant reductions in sperm concentration, motility, and total motile sperm in men from Columbia, Missouri, compared with men in New York City, Minneapolis, and Los Angeles. In an in-depth follow-up study comparing variables between the Columbia and Minneapolis men, the researcher discovered that the Missouri group had had higher exposure to agricultural pesticides. Further, men with low sperm counts were more likely to have higher urine metabolite levels of the pesticides alachlor, atrazine, metolachlor, and diazinon.

Another geographically based study, INUENDO, investigates risks to human fertility from persistent environmental organochlorines. The European Commission project centers on Arctic populations including Swedish fishermen and the Inuit of North America and Greenland, whose exposure to persistent organic pollutants such as PCBs and DDT metabolites are among the highest in the world. “There are many indications from animal studies and from wildlife studies, but very few indications from human studies telling us whether we have a problem or not,” says Bonde, who serves as coordinator of INUENDO.

“The basic idea [behind INUENDO] was to go to places in the world where we know that people have high level of exposures to substances that are suspected to cause these effects in fertility,” says Bonde. “That’s the reason we went to Greenland and to Sweden, where fishermen are known to have very high exposure levels; we have other populations that have lower levels of exposures, so we have contrasts of exposure.” Results published in March 2006 in *Human Reproduction* suggested a longer time to pregnancy related to serum concentrations of PCB and DDE in mothers and fathers. Additional results published in the May 2006 *EHP* suggested an altered sex ratio of offspring (fewer boys than would otherwise be expected) related to PCB and DDE exposures.

Exploring multicompound exposures is yet another challenge in environmental epidemiology. “Individuals are exposed to many different phthalates, a variety of persistent and nonpersistent pesticides, different patterns of PCB congeners, as well as other chemicals,” says Hauser. “How do we take all that information, based on the chemical assessment in urine or in blood, and use that to assign exposure for that individual to ten, or twelve, or many more different compounds?” he says. In the April 2006 issue of *EHP*, Hauser’s group described evidence suggesting a relationship between PCBs and phthalates and human sperm motility, possibly due to PCBs’ inhibiting a key enzyme in phthalate metabolism.

Genes themselves offer another platform for investigation. Hugh Taylor, director of the Yale Center for Research in Reproductive Biology, leads a team investigating the role of estrogen-regulated *Hox* genes that direct uterine development. The researchers initially focused on DES effects and discovered that the compound alters expression of the *Hoxa10* gene in mice, affecting the tissue type that grows in the uterus, cervix, and vagina. Effects were triggered only with exposure during development, but not during adulthood, and later experiments revealed that the pesticide methoxychlor had similar effects.

“The important thing is that these agents really seem to imprint the expression pattern, even long after the agent is removed or there’s no longer an exposure,” says Taylor. “When we have a clear-cut animal model and know the genes that are affected, we can start to think about evaluating that exposure by looking for changes in the gene expression earlier and see if it has a significant effect rather than waiting a whole generation.”

This is a goal of research in epigenetics, the study of how genetic messages may be edited through methylation or other means without changing the actual DNA sequence. For example, Rebecca Sokol and colleagues at the University of Southern California are currently investigating whether DNA methylation in sperm might serve as a bio-marker of environmental exposure and a means of assessing male fertility. Additionally, preliminary work at Washington State University and at the NIEHS indicates that an epigenetic event in one generation can “reprogram” the germline and affect later generations. In essence, the exposures of one’s great-grandparents could still matter today.

## Expanding Understanding

Previous generations’ exposures would be useful information to have, according to Hunt. “What we really need is data on generations ago, and we simply don’t have that data,” she says. “We have to wait a generation to see. We have to wait until . . . young exposed males grow up to the point where we can assess sperm counts.”

This will require prospective studies to determine early exposures. “If you want to look at fertility—and it’s difficult to do—you ideally would want to do a study in which you start assessing environmental exposures preconception,” says Hauser. “You’d have to identify couples who are thinking of trying to conceive and try to understand their environmental exposures, and then follow them forward in time.”

According to Alison Carlson, a senior fellow at The Collaborative on Health and the Environment (CHE) in Bolinas, California, another need is very basic: tracking the incidences of infertility and common known causes. “For us to try to make headway studying environmental influences on fertility, it’s really hard when we don’t have good baseline data,” she says. “We don’t know the real incidence and prevalence rates of premature ovarian failure and polycystic ovarian syndrome and lots of other end points that people study. We don’t know what they are, so how can we study trends and the environmental contributions?” she asks.

A thorough exploration of environmental effects on fertility will require the expertise of demographers, epidemiologists, clinicians, biologists, wildlife researchers, geneticists, molecular biologists, exposure assessment specialists, toxicologists, and others—and discussion requires someone “to set the table,” says Carlson. A February 2005 workshop titled “Understanding Environmental Contaminants and Human Fertility Compromise: Science and Strategy” demonstrated multidisciplinary fervor for investigation, and a more in-depth conference, the “Summit on Environmental Challenges to Reproductive Health and Fertility,” cosponsored by CHE and the University of California, San Francisco, is scheduled for 28–30 January 2007. “Reproduction is such a human, deep-seated, deeply psychically coded thing,” says Carlson. “It’s hard not to care about fertility compromise.”

## Figures and Tables

**Figure:**
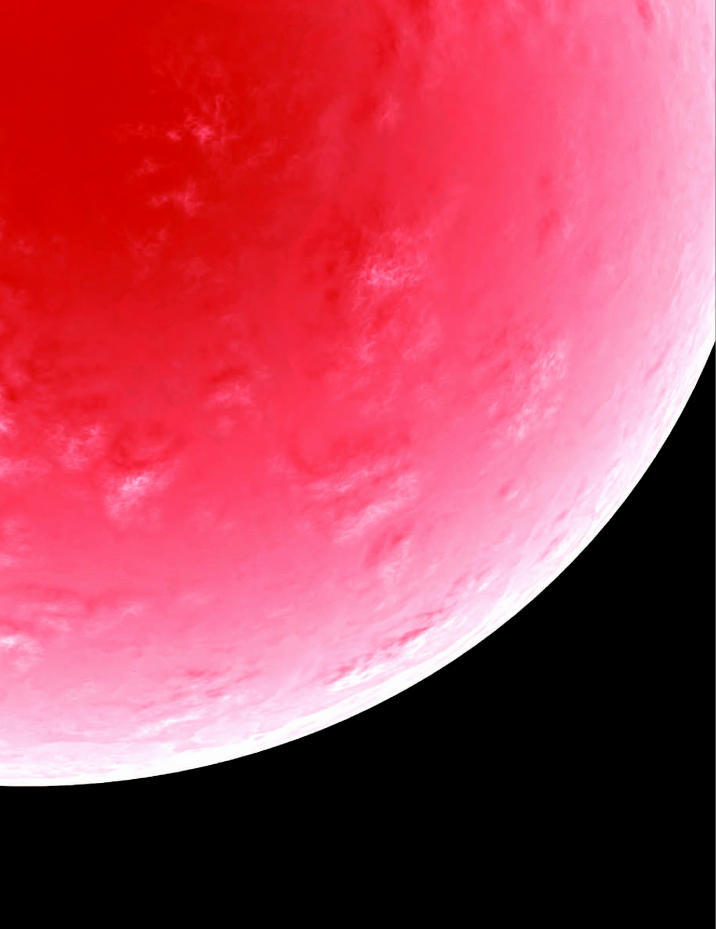


**Figure:**
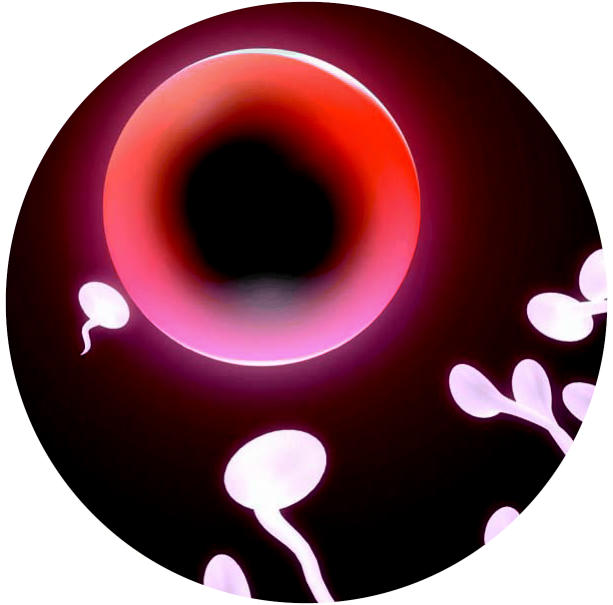
Her side Female factors in infertility stem from ovulation problems, thyroid irregularities, polycystic ovarian syndrome, and fallopian tube obstruction. A trend among women to delay starting a family also has impacted fertility rates.

**Figure:**
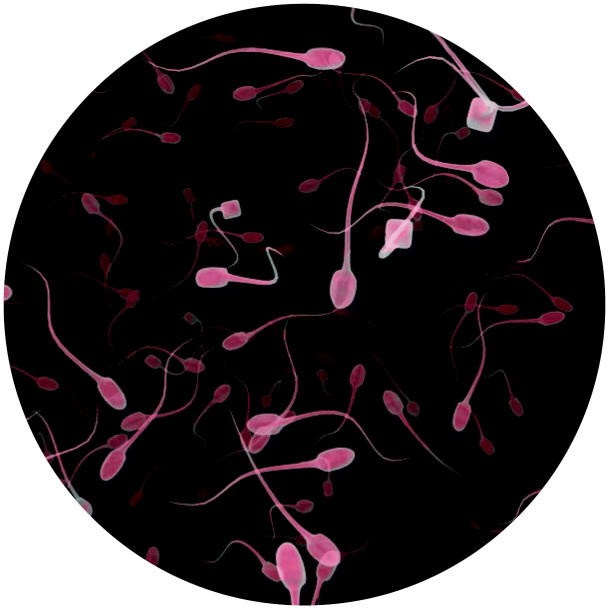
His side Male infertility can arise from factors such as low sperm count and sperm abnormalities including altered morphology and low motility. Up to 10% of infertility cannot be explained medically.

**Figure:**
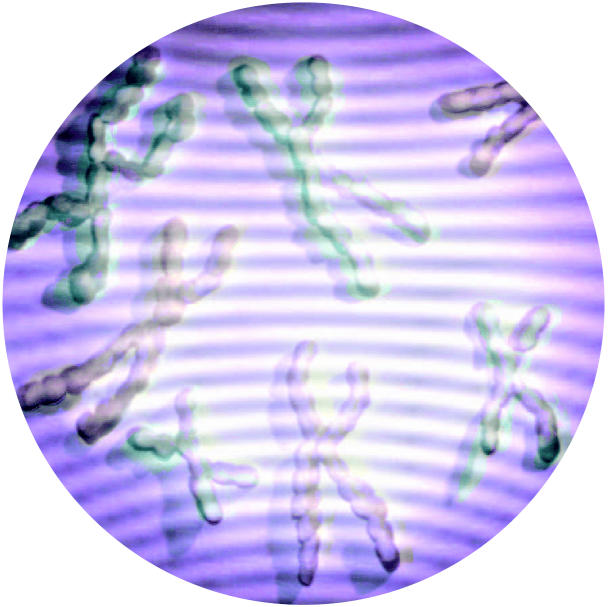
The wild side Animal and wildlife studies of reproductive health effects, including mouse aneuploidy data, may help inform knowledge of human effects. Although the reproductive system is highly conserved across species, differences in exposure, metabolism, and anatomy make direct interspecies comparisons impossible.

**Figure:**
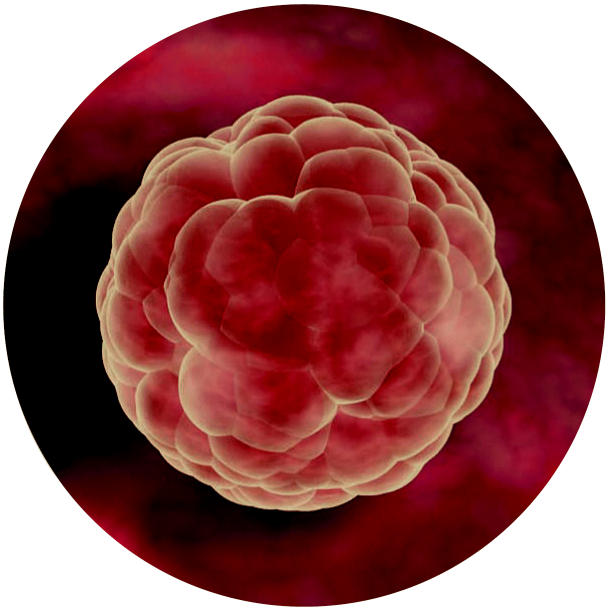
A view inside Understanding that a person’s reproductive health can be linked to the very earliest of exposures, possibly even paternal or maternal exposures prior to conception, points up the critical need to elucidate the health effects of environmental chemicals.

