# Erratum

**Published:** 2006-12

The November 2006 Focus article “Fertile Grounds for Inquiry: Environmental Effects on Human Reproduction” [ Environ Health Perspect 114:A644–A649 (2006)] contains two incorrect citations. Swan et al.’s review of declining human sperm counts (cited p. A647) appeared in the November, not October, 1997 issue of *EHP*, and Hauser et al. published their findings on PCBs, phthalates, and human sperm motility (cited p. A649) in the April 2005, not 2006, issue of *EHP. EHP* regrets the errors.

