# Erratum

**Published:** 2007-04

The November 2006 Focus article “Fertile Grounds for Inquiry: Environmental Effects on Human Reproduction” [ Environ Health Perspect 114:A644–A649 (2006)] contains a potentially misleading typo on page A646. The National Survey on Family Growth defined fertility as a married woman being able, not unable, to become pregnant within 12 months.

*EHP* regrets the error.

